# Robust brain parcellation using sparse representation on resting-state fMRI

**DOI:** 10.1007/s00429-014-0874-x

**Published:** 2014-08-26

**Authors:** Yu Zhang, Svenja Caspers, Lingzhong Fan, Yong Fan, Ming Song, Cirong Liu, Yin Mo, Christian Roski, Simon Eickhoff, Katrin Amunts, Tianzi Jiang

**Affiliations:** 1 Brainnetome Center, Institute of Automation, Chinese Academy of Sciences, Beijing, 100190 China; 2 National Laboratory of Pattern Recognition, Institute of Automation, Chinese Academy of Sciences, Beijing, 100190 China; 3 Institute of Neuroscience and Medicine (INM-1), Research Centre Juelich, 52425 Juelich, Germany; 4 Institute for Clinical Neuroscience and Medical Psychology, Heinrich-Heine-University Düsseldorf, 40225 Düsseldorf, Germany; 5 C. and O. Vogt Institute for Brain Research, Heinrich-Heine-University Düsseldorf, 40225 Düsseldorf, Germany; 6 Queensland Brain Institute, The University of Queensland, St Lucia, QLD 4072 Australia; 7 The First Affiliated Hospital of Kunming Medical University, Kunming, 650032 People’s Republic of China

**Keywords:** Resting state, Functional connectivity, Robust brain parcellation, Medial frontal cortex, Parietal operculum, Sparse representation

## Abstract

Resting-state fMRI (rs-fMRI) has been widely used to segregate the brain into individual modules based on the presence of distinct connectivity patterns. Many parcellation methods have been proposed for brain parcellation using rs-fMRI, but their results have been somewhat inconsistent, potentially due to various types of noise. In this study, we provide a robust parcellation method for rs-fMRI-based brain parcellation, which constructs a sparse similarity graph based on the sparse representation coefficients of each seed voxel and then uses spectral clustering to identify distinct modules. Both the local time-varying BOLD signals and whole-brain connectivity patterns may be used as features and yield similar parcellation results. The robustness of our method was tested on both simulated and real rs-fMRI datasets. In particular, on simulated rs-fMRI data, sparse representation achieved good performance across different noise levels, including high accuracy of parcellation and high robustness to noise. On real rs-fMRI data, stable parcellation of the medial frontal cortex (MFC) and parietal operculum (OP) were achieved on three different datasets, with high reproducibility within each dataset and high consistency across these results. Besides, the parcellation of MFC was little influenced by the degrees of spatial smoothing. Furthermore, the consistent parcellation of OP was also well corresponding to cytoarchitectonic subdivisions and known somatotopic organizations. Our results demonstrate a new promising approach to robust brain parcellation using resting-state fMRI by sparse representation.

**Electronic supplementary material:**

The online version of this article (doi:10.1007/s00429-014-0874-x) contains supplementary material, which is available to authorized users.

## Introduction

Resting-state fMRI (rs-fMRI) has been widely used to explore the functional coupling between distinct brain regions by calculating low-frequency spontaneous fluctuations in the time series, i.e., functional connectivity (Biswal et al. 1995; Fox and Raichle 2007; Buckner et al. 2013; Song and Jiang 2012). Functional connectivity has been a powerful tool to identify the resting-state networks (Greicius et al. 2003; Tomasi and Volkow 2012; Damoiseaux et al. 2006). Recently, rs-fMRI has also been exploited to delineate distinct subregions within a larger brain region based on differential patterns of functional connectivity (Craddock et al. 2012; Kim et al. 2010; Nelson et al. 2010; Yeo et al. 2011; Shen et al. 2010; Deen et al. 2011). As we know, the functional connectivity could be influenced by various artifacts in rs-fMRI data including physiological artifacts (Birn et al. 2008), transient head motion (Van Dijk et al. 2012), different scanning conditions (Patriat et al. 2013) and preprocessing procedures (Van Dijk et al. 2010; Satterthwaite et al. 2013). Hereby, these artifacts might also have impacts on the parcellation results. Generally, there are three approaches proposed to reduce the impact of noise during the parcellation procedures. The first is to average the connectivity profiles (Deen et al. 2011; Yeo et al. 2011) or similarity matrices across subjects (Craddock et al. 2012), which, however, eliminates inter-individual variability, which has been widely reported in both structure and function of the human brain (Mueller et al. 2013; Rademacher et al. 2001; Zilles and Amunts 2013). The second is to employ spatial constraints to improve the stability of parcellation (Craddock et al. 2012), which might bias the results towards spherical-shaped clusters. Another approach is to remove the noisy edges lying between clusters by constructing a sparse similarity matrix, for instance the KNN graph (Shen et al. 2010; von Luxburg 2007). But the KNN graph method requires a global sparsity parameter, which is often difficult to determinate (Nadler and Galun 2006) and could significantly affect the performance of parcellation (Shen et al. 2010). Thus, a more efficient sparse technique is required, which could generate robust brain parcellation by guaranteeing the stability of parcellation and retaining the individual variability at the same time.

The sparse representation theory (Elad 2010) has been widely employed in the classification of face, natural and medical images (Wright et al. 2009, 2010; Su et al. 2012; Wee et al. 2014; Mairal et al. 2008). Recently, it also has been proposed for data clustering and achieved robustness on high-dimensional data (Elhamifar and Vidal 2013), which construct a sparse similarity graph based on the sparse representation coefficients and employ the spectral clustering to cluster local subspaces (Elhamifar and Vidal 2009). Instead of identifying the linear dependence relations between each pair of variables, sparse representation employs the multivariate regression model to characterize the unique contribution of each point to the objective point. In addition to the self-representation model, an extra sparsity constraint on the representation coefficients is emphasized to identify the most relevant variables. Consequently, noise effects can be reduced (Elad and Aharon 2006; Elhamifar and Vidal 2013) and the signals may be recovered (Elad 2010). More importantly, the sparse representation coefficients may identify the nearest subspaces for each point with minimum embedding dimensions (Elhamifar and Vidal 2013; Wang and Xu 2013), which gives hints to the local organization of data. Thus, the similarity matrix constructed based on these representation coefficients could be used for data clustering (Elhamifar and Vidal 2013; Vidal 2011). Additionally, with the approximately block diagonal form (Elhamifar and Vidal 2013), the similarity matrix could maintain a hierarchical consistency when different clusterings were performed. All these properties could be very helpful for rs-fMRI-based brain parcellation.

Here, we propose a brain parcellation method based on the sparse representation, which is robust to noise and preserves individual variability during brain parcellation. We tested the method on simulated, multi-site and different spatially smoothed rs-fMRI datasets. The robustness of the method was first tested on the simulated rs-fMRI data. To further assess the stability of the method, two different brain areas, i.e., the medial frontal cortex (MFC, including SMA and pre-SMA) and parietal operculum (OP), were parcellated on multi-site rs-fMRI datasets. The parcellation of MFC with a clear segregation between SMA and pre-SMA has been widely used as a validation for parcellation methods (Johansen-Berg et al. 2004; Klein et al. 2007), especially on rs-fMRI data (Kim et al. 2010; Ryali et al. 2013; Crippa et al. 2011; Nanetti et al. 2009). Area OP, on the other hand, has been widely accepted as a heterogeneous region (Keysers et al. 2010; Zu Eulenburg et al. 2013; Burton et al. 2008), with cytoarchitectonic mapping of this region (Eickhoff et al. 2006a) available as a representation of its microstructure and a clear somatotopic organization (Eickhoff et al. 2007) among its subdivisions. Thus, we first subdivided MFC on multi-site datasets to evaluate the consistency across different datasets and on differently smoothed datasets to study the influence of smoothing conditions. Then, we parcellated OP using rs-fMRI data and compared its functional parcellation with the cytoarchitectonic subdivisions (Eickhoff et al. 2006a).

## Materials and methods

### Brain parcellation using sparse representation

**Fig. 1:**
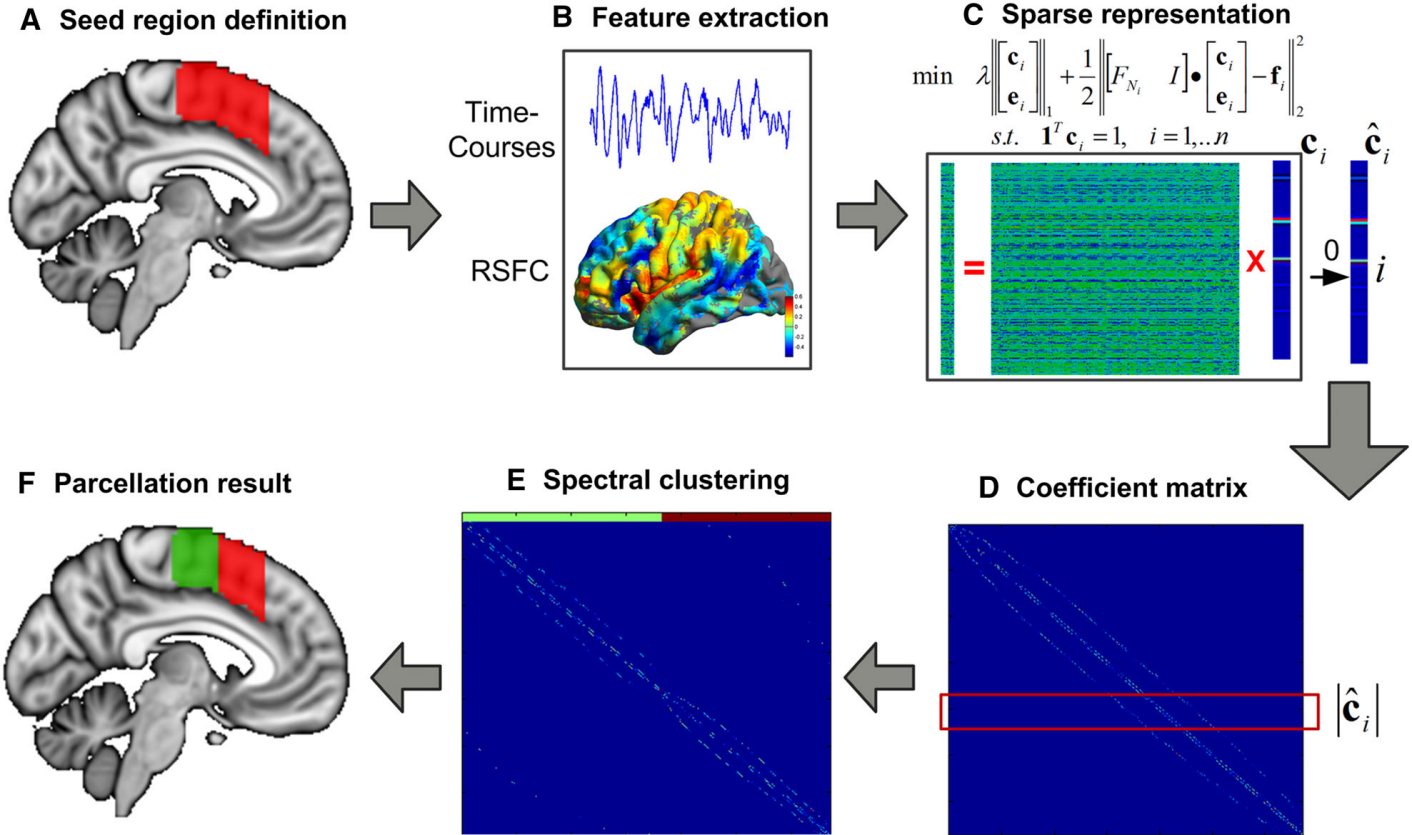
Brain parcellation scheme using sparse representation. After defining the seed region in the standard space (**a**), we generated the feature matrix by extracting time courses within the seed region or calculating the whole-brain functional connectivity patterns (**b**). For each seed voxel, the $\ell_{1}$-norm minimization problem was solved independently and its representation coefficient vector ${\mathbf{c}}_{i}$ was extended into ${\hat{\mathbf{c}}}_{i}$ through the insertion of a zero entry at the $i$-th row (**c**). Then, the absolute value of the coefficients $\left| {{\hat{\mathbf{c}}}_{i} } \right|$ was combined into a coefficient matrix, and a similarity matrix was constructed based on it (**d**). Finally, spectral clustering was applied to the similarity matrix (**e**), generating parcellation results for each individual (**f**)

The proposed parcellation scheme consisted of two key steps. First, sparse representation (SR) was employed to calculate the representation coefficients for each voxel by all the other voxels (Fig. 1a–c). Then, a similarity matrix was constructed based on these representation coefficients (Fig. 1d), and spectral clustering was applied to generate individual parcellation for each subject (Fig. 1e–f).

### Sparse representation

After defining a seed mask in the Montreal Neurological Institute (MNI) standard space (Fig. 1a), the time course of each voxel within the seed region was extracted from the rs-fMRI datasets (Fig. 1b), serving as the data matrix in sparse representation. Importantly, both the whole-brain functional connectivity patterns and the local time-varying BOLD signals may be employed as features in our method. Here, we focus on the use of the local time courses to illustrate the proposed method. Each voxel may be represented as a sparse linear combination of other voxels within the seed region (Fig. 1c). The linear representation was intrinsically sparse, because seed voxels were highly correlated with spatially neighboring voxels due to the averaging effect of BOLD signals and spatial smoothing. The sparse representation of each voxel was calculated by solving the convex $\ell_{1}$-norm minimization problem (Eq. 1).

1 $$\hbox{min} \,\left\| {{\mathbf{c}}_{i} } \right\|_{1} + \left\| {{\mathbf{e}}_{i} } \right\|_{1} \quad \begin{array}{*{20}c} {\begin{array}{*{20}c} {{\text{s}} . {\text{t}} .} & {\left\| {F_{{N_{i} }} {\mathbf{c}}_{i} + {\mathbf{e}}_{i} - {\mathbf{f}}_{i} } \right\|_{2} \le \varepsilon } \\ \end{array} } & {i = 1, \ldots n} \\ \end{array}$$

where ${\mathbf{f}}_{i} \in R^{d}$ is the feature vector of voxel *v*_*i*_ with a unit norm $\left\| {f_{i} } \right\|_{2} = \sqrt {\sum\nolimits_{j} {\left| {f_{ji} } \right|^{2} } } = 1$, $F_{{N_{i} }} \in R^{d \times (n - 1)}$ is the residual feature matrix within the seed region by eliminating voxel *v*_*i*_, *ε* is a small real number to control the accuracy of the liner representation, ${\mathbf{c}}_{i} \in R^{n - 1}$ is the representation coefficient vector with the summation constraints ${\mathbf{1}}^{\text{T}} {\mathbf{c}}_{i} = \sum\nolimits_{j} {c_{ji} } = 1$ which could accelerate the convergence process of the Lasso problem, and the $\ell_{1}$-norm was defined as $\left\| {{\mathbf{c}}_{i} } \right\|_{1} = \sum\nolimits_{j} {\left| {c_{ji} } \right|}$. Here, the feature vector is referring to the time course of each voxel, with *n* denoting the total number of voxels within the seed region and *d* denoting the number of time points.

The above objective function (Eq. 1) can then be converted into an equivalent Lagrangian function:

2 $$\hbox{min} \lambda \left\| {\left[ {\begin{array}{*{20}c} {{\mathbf{c}}_{i} } \\ {{\mathbf{e}}_{i} } \\ \end{array} } \right]} \right\|_{1} + \frac{1}{2}\left\| {\left[ {\begin{array}{*{20}c} {F_{{N_{i} }} } & I \\ \end{array} } \right] \cdot \left[ {\begin{array}{*{20}c} {{\mathbf{c}}_{i} } \\ {{\mathbf{e}}_{i} } \\ \end{array} } \right] - {\mathbf{f}}_{i} } \right\|_{2}^{2} \quad {\text{s}} . {\text{t}}.\quad 1^{\text{T}} {\mathbf{c}}_{i} = 1,\quad i = 1, \ldots n$$

where the sparsity parameter *λ* is a tradeoff between the accuracy of the linear expression and the sparsity of the coefficient vector. In addition, each coefficient vector **c**_*i*_ is extended into an *n*-dimensional vector ${\hat{\mathbf{c}}}_{i}$ by inserting a zero entry at the *i*-th row, which represents the neighborhood relationship between voxel *v*_*i*_ and the remaining voxels in the seed region. To solve the $\ell_{1}$-minimization problem (Eq. 2), we used the basis pursuit denoising homotopy (BPDN homotopy) method (http://www.eecs.berkeley.edu/~yang/software/l1benchmark/l1benchmark.zip; Yang et al. 2010), which starts at the trivial solution ${\mathbf{x}}_{0} = {\mathbf{0}}$, and successively builds a sparse solution by adding or removing elements from its active set until the representation error term was satisfied (Donoho and Tsaig 2008). We chose this method because of its high computational efficiency and robustness against corruption (Yang et al. 2010), which could solve the sparse representation problem in less than 2 h for the whole brain.

### Spectral clustering

After solving the sparse representation equation for each seed voxel (Fig. 1 c), the coefficient vectors were combined into a coefficient matrix $C = [{\hat{\mathbf{c}}}_{1}^{\text{T}} ;{\hat{\mathbf{c}}}_{2}^{\text{T}} ; \ldots ;{\hat{\mathbf{c}}}_{n}^{\text{T}} ]$ with zero-diagonal elements (Fig. 1d). A directed graph was constructed based on the coefficient matrix, with each node denoting a seed voxel *v*_*i*_ who is only adjacent to the voxels with nonzero entries in its coefficient vector ${\hat{\mathbf{c}}}_{i}^{\text{T}}$, and the weight of edges defined by the absolute value of its representation coefficients $\left| {{\hat{\mathbf{c}}}_{i} } \right|$. The coefficient matrix C, with zero-diagonal elements and unit sum for each row, represents the transition matrix of random walk on the graph. Specifically, each element $\hat{c}_{ij}$ represents the probability of transition from voxel *v*_*i*_ to voxel *v*_*j*_. Thus, the element $\sum\nolimits_{k} {\hat{c}_{ik} \hat{c}_{jk} }$ in $C \cdot C^{\text{T}}$ represents the joint probability of transitions from voxel *v*_*i*_ and voxel *v*_*j*_ to the same end, which also represents the probability that voxel *v*_*i*_ and voxel *v*_*j*_ were represented by the same other voxels or located in the same subspace. But, the effect of “hub” representation voxels that appear in the majority of representation equations should be avoided. In other words, the contribution of each voxel to each representation should be normalized by its summation in all representations. Therefore, the final similarity matrix was defined as $W = C \cdot E^{ - 1} \cdot C^{\text{T}}$, where *E* was the diagonal matrix saving the column summations of the coefficient matrix *C*. The similarity matrix *W* could be applied to spectral clustering to generate the final parcellation results (Fig. 1e–f).

In spectral clustering, a similarity graph was first built, with each node denoting a seed voxel and the voxel-to-voxel similarity matrix defining the weight of the edges. It is worth mentioned that the spectral clustering used here is different from the spectral reordering method (Johansen-Berg et al. 2004), which requires a manual definition of the boundaries between clusters. Spectral clustering is intended to calculate the spectral embedding of the data, which is actually a nonlinear dimensionality reduction process (von Luxburg 2007). First, the Laplacian matrix was calculated as *L* = *D* − *W*, where *D* was a diagonal matrix saving the degree of each node (i.e., the row sum). Second, the generalized eigenvalue problem $Lu = \mu Du$ was solved, with its first few eigenvectors $u_{i} ,\;i = 1, \ldots ,k$ saved in a matrix *U* as a low-dimensional representation of the data, where *k* was specified by the predefined cluster number. Last, classical clustering methods, such as k-means clustering (Ng et al. 2002) or orthogonal projection (Shi and Malik 2000), were applied to the spectral embedding matrix *U*. Here, we used the *k*-means clustering during the implementation of spectral clustering.

The proposed method generates individual parcellation results for each subject with the predefined cluster numbers (Fig. 1f). For real rs-fMRI data, an additional group-level parcellation procedure was performed on each dataset separately. Specifically, first, the parcellation results on each subject was aligned with each other to have the same labeling scheme. Then, a population probabilistic map of each cluster was calculated by counting the percentage among the subjects who had the same labels at the specific voxels. Last, these probabilistic maps were merged into a maximum probability map (MPM) based on the majority rule (each voxel is assigned to the cluster with the highest probability).

### Seed regions

The medial frontal cortex (MFC) and the parietal operculum (OP) were chosen as the seed regions. They were both defined in Montreal Neurological Institute (MNI) space and resliced into 3 mm using FLIRT (Jenkinson and Smith 2001). The MFC, including the supplementary (SMA) and pre-supplementary motor area (pre-SMA), was manually drawn in the MNI152 brain image, extending from *y* = −22 to *y* = 30, with a short distance above the cingulate sulcus (Johansen-Berg et al. 2004; Eickhoff et al. 2011). The parietal operculum, consisting of four subregions in each hemisphere, was extracted as the MPM image based on the cytoarchitectonic subdivisions (Eickhoff et al. 2006a) using the Anatomy toolbox (Eickhoff et al. 2005). The rs-fMRI time course of each seed voxel was extracted from the preprocessed fMRI datasets and employed as the feature matrix in sparse representation.

### Data acquisition and preprocessing

We acquired three different resting-state fMRI datasets with eyes closed from a total of 93 healthy right-handed participants. Detailed information of these subjects was listed in Table 1. All subjects provided written informed consent to the study protocol as approved by the local ethics committee. The subjects were instructed to rest with their eyes closed, relax their minds, and remain as motionless as possible during the scanning. The first two datasets were acquired from two different Chinese populations using the same Philips Achieva 3.0 T MRI scanner. The first dataset consisted of 29 subjects [16 males; age range = 20–36 years, mean age = 25.0, standard deviation (SD) = 4.35]. The second dataset consisted of 32 subjects (14 males; age range = 22–34 years, mean age = 26.0, SD = 2.1). A total of 240 volumes, each covering the entire brain including the cerebellum with 33 axial slices, were acquired using gradient-echo echo planar imaging (EPI) sequence [repetition time (TR) = 2,000 ms, echo time (TE) = 30 ms, field of view (FOV) = 220 × 220 mm^2^, matrix = 64 × 64, slice thickness = 4 mm, gap = 0.6 mm, flip angle = 90°]. A structural scan was also acquired for each participant, using a T1-weighted 3D turbo field echo (TFE) sequence (TR = 8.2 s, TE = 3.8 ms, FOV = 256 × 256 mm^2^, matrix = 256 × 256, number of slices = 188, slice thickness = 1 mm, no gap, flip angle = 7°).

**Table 1:** Detailed information of the subjects from the three rs-fMRI datasets

Datasets	Scanner	Populations	Subjects	Age range	Gender
Dataset 1	3.0 T Philips	Chinese Bai	29	20–36, mean 25.0	16 males
Dataset 2	3.0 T Philips	Chinese Han	32	22–34, mean 26.0	14 males
Dataset 3	3.0 T Siemens	German	32	22–39, mean 29.0	14 males

Using a Siemens Tim-TRIO 3.0 T MRI scanner, the third dataset was acquired from 32 German participants (14 males; age range = 22–39 years, mean age = 29.0, SD = 4.82), selected from a sample of 100 subjects at the Research Centre Jülich used in the following studies (Kellermann et al. 2013; Jakobs et al. 2012; Zu Eulenburg et al. 2012; Cieslik et al. 2013; Rottschy et al. 2013) to match the age and gender of the other two datasets. For each subject, 300 resting-state EPI images were acquired using BOLD contrast [gradient-echo EPI pulse sequence, TR = 2.2 s, TE = 30 ms, flip angle = 90°, in plane resolution = 3.1 × 3.1 mm^2^, 36 axial slices (3.1 mm thickness) covering the entire brain]. A structural scan was also acquired for each participant, using a T1-weighted 3D magnetization-prepared rapid acquisition with gradient-echo (MPRAGE) sequence (176 axial slices, TR = 2.25 s, TE = 3.03 ms, FOV = 256 × 256 mm^2^, flip angle = 9°, final voxel resolution: 1 mm × 1 mm × 1 mm).

All three rs-fMRI datasets were preprocessed using the same script as described in the 1000 Functional Connectome Project (http://www.nitrc.org/projects/fcon_1000) (Biswal et al. 2010). The preprocessing steps included: (1) discarding of the first ten volumes in each scan series for signal equilibration, (2) performing slice timing correction and motion correction, (3) removing the linear and quadratic trends, (4) band-pass temporal filtering (0.01 Hz < *f* < 0.08 Hz), (5) spatial smoothing using 6-mm full-width at half-maximum (FWHM) Gaussian kernel, (6) performing nuisance signal regression [including white matter (WM), cerebrospinal fluid (CSF), the global signal, and six motion parameters], and (7) resampling into Montreal Neurological Institute’s (MNI) space with concatenated transformations from the mean functional volume to the individual anatomical volume and spatial normalization of the individual anatomical volume to the MNI152 brain template. Finally, a four-dimensional time-series dataset in standard MNI space was obtained for each subject after preprocessing. No participant exhibited head motion of more than 1.5-mm translation or 1.5° angular rotation. In addition, to generate differently smoothed datasets, the second dataset was also spatially smoothed using different Gaussian kernels (i.e., unsmoothed, FWHM = 4, 6 and 8 mm).

### Simulated rs-fMRI datasets

**Fig. 2:**
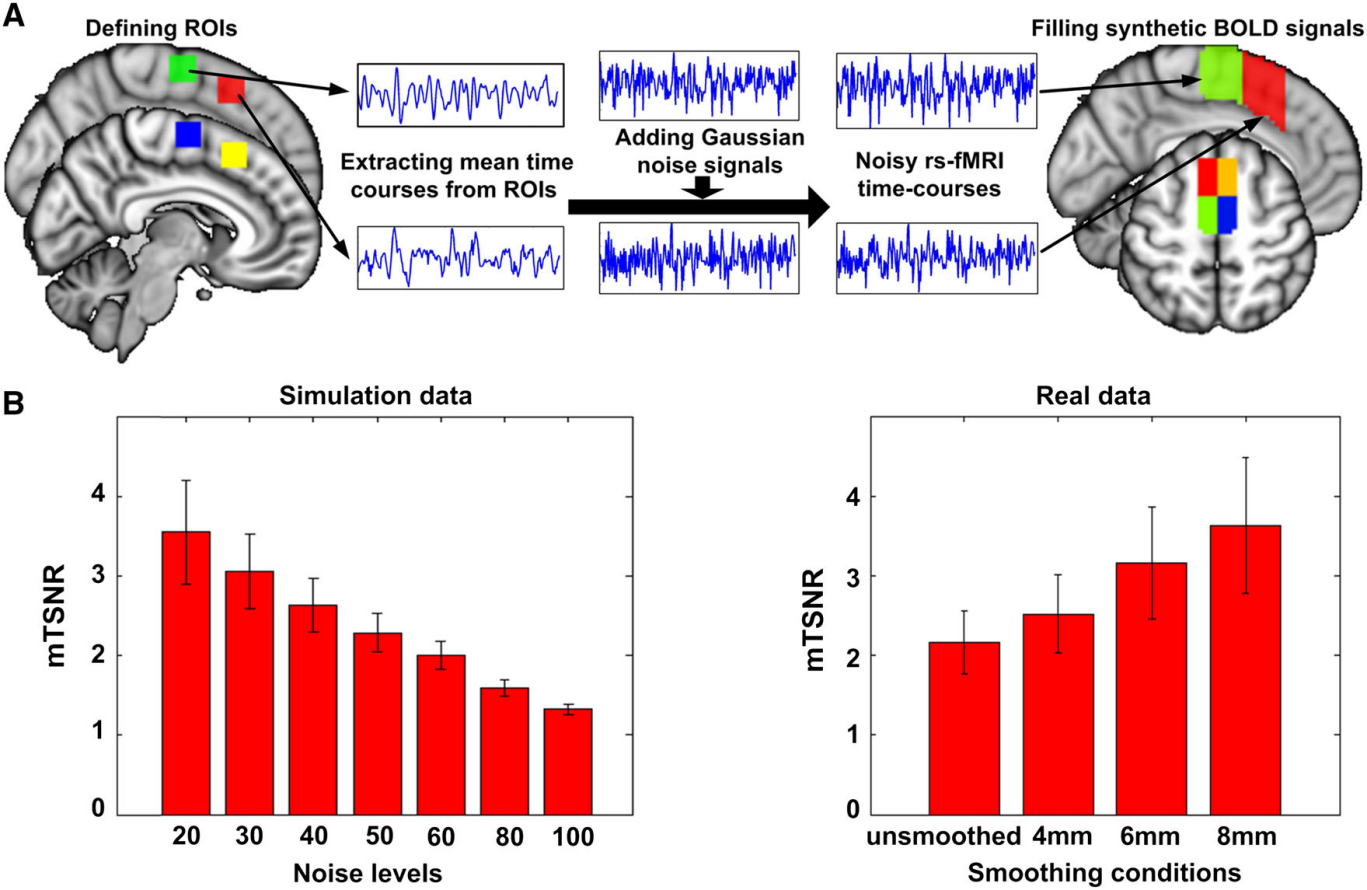
Generation of the simulated rs-fMRI datasets. Four subunits were defined within medial frontal cortex (MFC) and filled with noisy synthetic BOLD signals (**a**). MFC was manually separated into supplementary motor area (SMA) and pre-SMA in both hemispheres using a vertical line at *y* = 0 (Zilles et al. 1996). Each of the four subunits was filled with different source signals, and each voxel within a single subunit had different noise signals. First, a ROI was manually defined on each subunit, and its mean time course was extracted as the source signal for the synthetic data of the corresponding subunit. Then, different amounts of Gaussian noise were added throughout the entire seed region. Finally, the simulation data was generated by filling each subunit with the corresponding synthetic BOLD signals. The mean temporal signal-to-noise ratio (mTSNR) was calculated for each simulation data and compared with the real data using different smoothing conditions (**b**). The *error bars* represent the SDs of mTSNR across a group of subjects. Generally, the low noisy simulation data (SD = 20–60) had comparable TSNR with the real data, while the high noisy simulation data (SD = 80 and 100) had worse TSNR than the real data

The simulated rs-fMRI datasets were generated based on the preprocessed rs-fMRI data selected from the second dataset, filling the bilateral medial frontal cortex (MFC) with synthetic BOLD signals instead of original time-varying signals (Fig. 2). As preparation, the seed region was first manually separated into supplementary motor area (SMA) and pre-SMA in both hemispheres using a vertical line at *y* = 0 as the boundary (Zilles et al. 1996; Picard and Strick 1996). Then, the simulated dataset was generated by the following steps: (1) defining a region of interest (ROI) on each subunit, i.e., ROI1/ROI2 was a 3 × 3 × 3 cube centered at (±9, −6, 64) in SMA, and ROI3/ROI4 was a 3 × 3 × 3 cube centered at (±8, 22, 50) in pre-SMA; (2) extracting the mean time courses from each of the four ROIs and using these time courses as the source signals for the synthetic data in the corresponding subunit; (3) adding different amounts of Gaussian noise throughout the entire seed region. As a result, each of the four subunits was filled with different synthetic noisy time courses, i.e., different source signals among the four subunits and different noise signals within each subunit.

We constructed seven sets of the simulated rs-fMRI data, each consisting of ten virtual subjects, contaminated with different noise levels, ranging from fairly low levels (the SD of additional noise is 20) to relatively high levels (SD = 100). Notably, the noise has been spatially smoothed with a three-voxel Gaussian kernel. Additionally, we calculated the mean temporal signal-to-noise ratio (mTSNR) (Murphy et al. 2007) to evaluate the SNR of the simulation data.

3 $${\text{TSNR}}_{i} = \frac{{\frac{1}{T}\sum\nolimits_{k} {f_{ik} } }}{{\sqrt {\frac{1}{T}\sum\nolimits_{k} {\left( {f_{ik} - \frac{1}{T}\sum\nolimits_{{f_{ik} }} {f_{ik} } } \right)^{2} } } }},\quad {\text{mTSNR}} = \frac{1}{N}\sum\limits_{i} {{\text{TSNR}}_{i} }$$

where *f*_*ik*_ stands for the BOLD signals of voxel *v*_*i*_ at the *k*-th time point and TSNR_*i*_ stands for the TSNR of voxel *v*_*i*_, with *T* denoting the total number of time points and *N* denoting the total number of voxels within the seed region. Our simulation data had comparable SNR with the real data using different smoothing conditions, as shown in Fig. 2. For the simulation data with a low noise level (SD = 20), the TSNR was comparable with the real data spatially smoothed with a FWHM = 8 mm kernel. For the simulation data with a relatively high noise level (SD = 60), the TSNR was comparable with the unsmoothed real data. As for very high noise levels, i.e., SD = 80 or 100, the TSNR of the simulation data was lower than that for the real rs-fMRI data.

### Sparsity parameter selection

The sparsity parameter *λ* in the sparse representation equation (Eq. 2) controls the number of nonzero entries in the representation coefficients. It consequently controls the sparsity of the similarity graph constructed based on them. When *λ* was too small, i.e., close to zero, the similarity graph could be very dense with each voxel represented by all other voxels. When *λ* was too large, i.e., some constant larger than one, the similarity graph would be too sparse to be a connected graph with each voxel only represented by one single voxel. Theoretically, there is a stable range for this parameter (Wang and Xu 2013). To assess the appropriate sparsity parameters, two different *λ* sequences were tested on the simulated rs-fMRI datasets. First, each value within the *λ* sequence [0, 0.0001, 0.001, 0.01, 0.1, 1, 10] was employed to evaluate the accuracy of MFC parcellation (on the simulation data), which showed that *λ* = 0.1 or 1 to be the most stable parameter. Next, the range [0.1, 1] was sampled with linearly equal steps with 0.1. We demonstrated that, when the *λ* value was within the range [0.1, 1], stable performance was achieved with very high robustness to noise. After determining a stable parameter range on simulated datasets, we only selected *λ* = 0.1 for real rs-fMRI datasets, but similar results were achieved when other parameters within the stable range were used (Fig. S6, see Supplementary Results 2 in Supplementary materials for a detailed explanation).

### Performance evaluation and group consistency

For the simulation data, the performance was evaluated through a comparison with the ground truth, which defined the vertical line *y* = 0 as the boundary (Zilles et al. 1996). Unfortunately, we had no access to the ground truth for real rs-fMRI data. In compensation, we evaluated the reproducibility of the parcellation results within each dataset, the consistency across multi-site datasets and the agreement across different smoothing conditions. The parcellation results were also compared with the cytoarchitectonic mapping of subdivisions (cyto-maps) when they were accessible. These indicators were all evaluated using the normalized mutual information (NMI, Eq. 4) (Lancichinetti and Fortunato 2009; Danon et al. 2005), ranging from 0 to 1, with 1 indicating the same parcellation with only differences in the sequence of labels, and 0 indicating totally different parcellation.

4 $${\text{NMI}} = \frac{I(X;Y)}{\hbox{min} (H(X),H(Y))} = \frac{{\sum\nolimits_{x} {\sum\nolimits_{y} {N_{xy} \log \frac{{N_{xy} \cdot N}}{{N_{x \cdot } N_{ \cdot y} }}} } }}{{\hbox{min} \left\{ {\sum\nolimits_{x} {N_{x \cdot } \log \frac{{N_{x \cdot } }}{N}} ,\;\sum\nolimits_{y} {N_{ \cdot y} \log \frac{{N_{ \cdot y} }}{N}} } \right\}}}$$

where *I*(*X*; *Y*) is the mutual information between the distributions of parameters *X* and *Y*, while *H*(*X*) and *H*(*Y*) are the entropies of the distributions for *X* and *Y*, respectively. Here, we used the minimum of the two entropies to normalize the mutual information. The NMI value could be calculated using the Contingency Table (Vinh et al. 2010), which records co-occurrence between any two clusters in two different parcellation results.

To evaluate the consistency among different datasets, the agreement between the MPMs of any two datasets was calculated using NMI (Eq. 4). To calculate the reproducibility of parcellation on each dataset, the entire group was randomly separated into two sub-groups, with one parcellation result for each sub-group, and the consistency between the two results was evaluated using NMI (Eq. 4). The entire procedure was repeated 100 times and the mean NMI value was calculated with higher values indicating better reproducibility of results between different sub-groups. As for the smoothing effects, the agreement of individual parcellation on different spatially smoothed data was evaluated using NMI (Eq. 4). The mean NMI value across subjects was calculated with higher values indicating low sensitivity to smoothing conditions for rs-fMRI data.

## Results

### Results on simulated rs-fMRI datasets

**Fig. 3:**
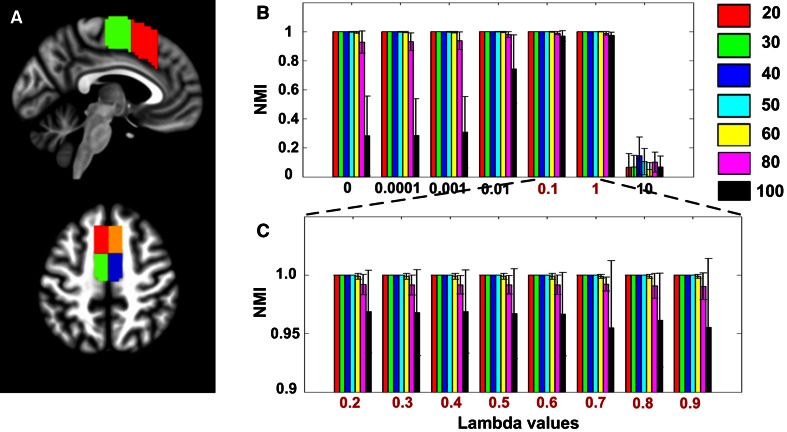
Performance of brain parcellation using sparse representation on the simulated rs-fMRI datasets. The simulation data were constructed to include four subunits within medial frontal cortex (**a**) and the parcellation method was tested on it with two *λ* sequences (**b**, **c**). Our method achieved high accuracy of parcellation on the low noisy data (SD = 20–60), but with a significant decrease on highly noisy data (SD = 80 and 100). However, a stable sparsity parameter range labeled with the *red color* could be identified which achieved highly stable performance on all noisy datasets. Each *column* in **b** corresponds to the accuracy of parcellation using parameters within the sequence [0, 0.0001, 0.001, 0.01, 0.1, 1, 10] and each *column* in **c** corresponds to using parameters within the sequence [0.2, 0.3, 0.4, 0.5, 0.6, 0.7, 0.8, 0.9]. The mean NMI scores across ten subjects was used to evaluate the accuracy of parcellation, with different colors indicating different noisy datasets, i.e., SD (noise) = 20, 30, 40, 50, 60, 80 and 100, and the *error bars* representing the SD of NMI values

Our method was tested on the simulation data by evaluating two types of performance (Fig. 3), including the accuracy of separating the predefined subunits and the ability of restraining noise effects. During parcellation, two different *λ* sequences were tested: the first one ranged from low sparsity (i.e., *λ* = 0.0001) to high sparsity (i.e., *λ* = 10) with logarithmic equal steps, and the second one focused on a local range [0.1, 1] with linear equal steps. As shown in Fig. 3, all parameters achieved high accuracy of parcellation on the low noisy data (SD = 20–60), but with a significant decrease on highly noisy data (SD = 80 and 100) which actually had worse TSNR than the real rs-fMRI data (Fig. 2b). More specifically, the method achieved unsatisfying performance in restraining noise when *λ* was too small (i.e., *λ* < 0.1), and failed in parcellation when *λ* was too large (i.e., *λ* > 1). However, a stable sparsity parameter range, i.e., the interval [0.1, 1], could be identified which achieved high accuracy of parcellation and high robustness to noise at the same time. Specifically, good performance was achieved on different noisy datasets with the mean NMIs >0.95 and SDs <0.1, when using each value in the second *λ* sequence (Fig. 3). Similar results were also shown when the cluster number was smaller than the ground truth, i.e., *K* = 2 and 3 (Fig. S3).

**Fig. 4:**
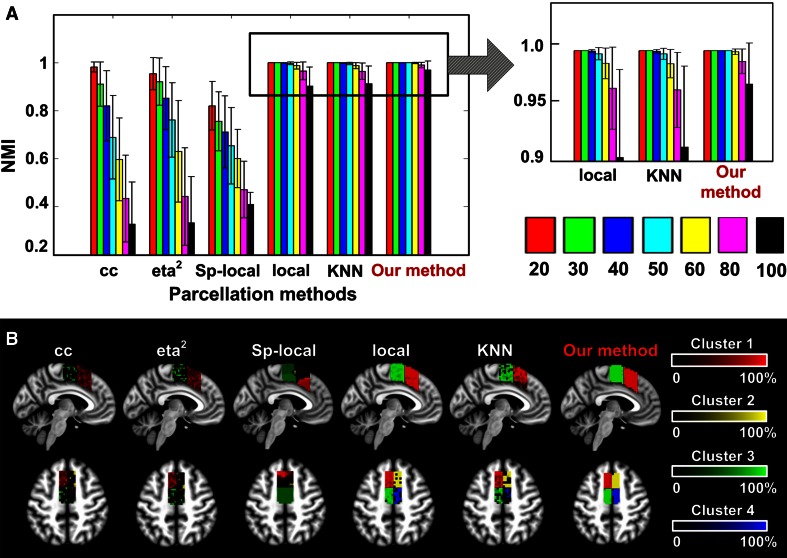
Comparing the performance with common parcellation methods on simulation data. Five commonly used similarity matrices were tested and compared with our method on different noisy datasets, i.e., SD (noise) = 20, 30, 40, 50, 60, 80 and 100. Our method achieved higher performance than all common methods on highly noisy data (SD = 80 and 100) and also showed higher robustness to noise. **a** Accuracy of parcellation evaluated through a comparison with the ground truth using normalized mutual information (NMI). **b** Sagittal (*x* = −4) and axial (*z* = 50) slice views of the overlapping maps of the parcellation results on the most noisy simulation dataset (SD = 100). cc, cross-correlation; local, using local time-varying BOLD signals; Sp-local, performing spatially constraints on the local matrix; KNN, constructing a KNN graph on the local matrix; Our method, performing sparse representation on local time-varying BOLD signals

We also compared the performance with commonly used similarity matrices, including cross-correlation (cc) (Chang et al. 2013; Kim et al. 2010; Bzdok et al. 2013), eta2 (Nelson et al. 2010; Kelly et al. 2012), spatially constrained (sp-local) (Craddock et al. 2012) and Gaussian-kernel weighted on the local time-varying BOLD signals (local) (Shen et al. 2010), and KNN graph built on the local time-varying BOLD signals (KNN) (Shen et al. 2010). As shown in Fig. 4, our method achieved the highest accuracy in separating the four subunits on all datasets and showed much higher robustness to noise (i.e., with lower reduction in NMI values and lower SD as the noise level increased). Besides, our method also achieved higher performance when the predefined cluster number was smaller than the ground truth, i.e., *K* = 2 and 3 (Fig. S4).

### Parcellation results on real rs-fMRI datasets

#### Parcellation of MFC on real rs-fMRI data

Medial frontal cortex was partitioned into the putative SMA and pre-SMA in both hemispheres with a slightly oblique boundary close to y = 0, consistent with previous studies (Nanetti et al. 2009; Zhang et al. 2012; Kim et al. 2010; Eickhoff et al. 2011). Highly consistent results were achieved on three different datasets with little influence of different spatially smoothing conditions. The following results were mainly based on local time courses, but similar conclusions were drawn when whole-brain connectivity patterns were used as features (see Supplementary Results 1 in Supplementary materials for a detailed explanation).

**Fig. 5:**
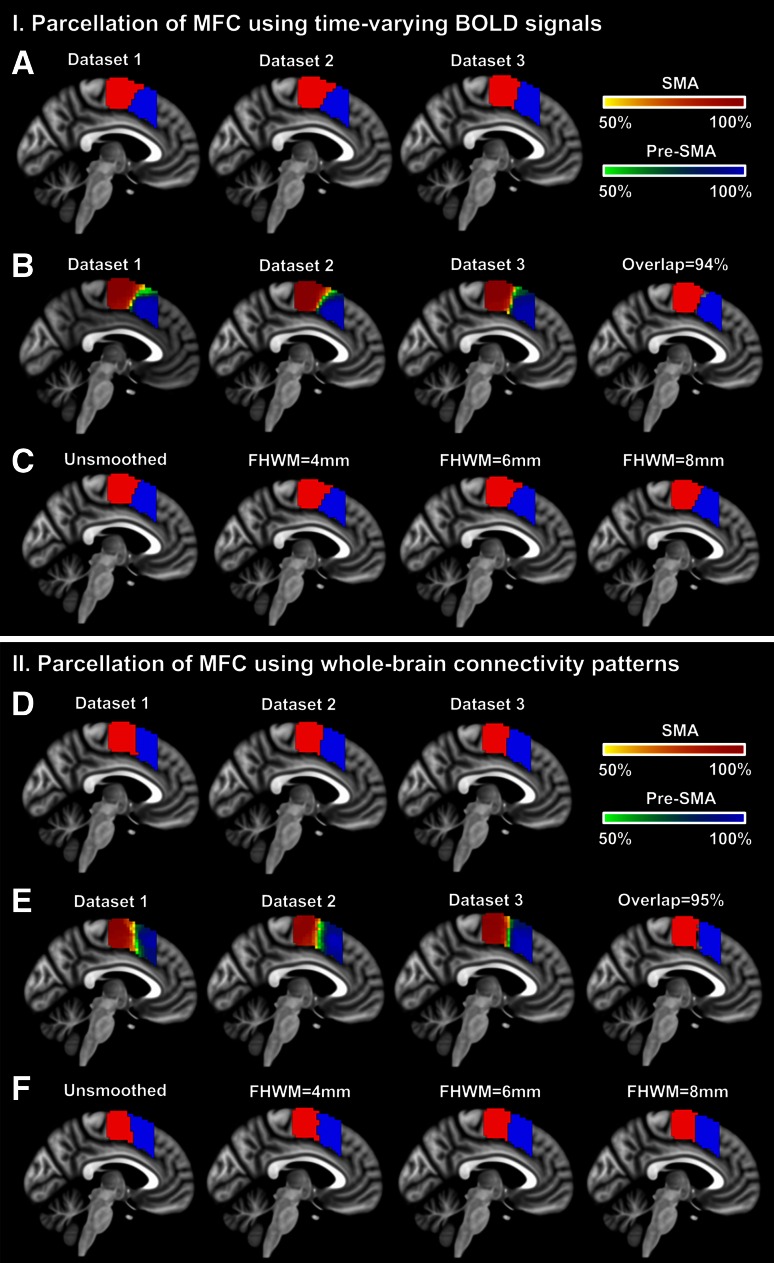
Parcellation of MFC on multi-site rs-fMRI datasets. MFC was partitioned into the putative SMA and pre-SMA using sparse representation on both time-varying BOLD signals (**a**–**c**) and whole-brain connectivity patterns (**d**–**f**). Consistent parcellation of MFC was achieved on three different datasets and on different spatially smoothed datasets. Additionally, similar results were generated when the two different features were used. The MPMs were calculated on each dataset separately, showing a high consistency with each other (**a**, **d**). The probability maps of the two clusters were thresholded at 50 % and displayed on the same image for each dataset (**b**, **e**). An ROI was extracted for each cluster with a probability threshold at 50 % and intersected among three datasets to calculate the overlapping maps. Consistent parcellation of MFC was achieved under different smoothing conditions (**c**, **f**), i.e., unsmoothed, FWHM = 4, 6 and 8 mm. All results were shown at the slice *x* = −4 (MNI coordinate), and overlapped on the MNI152 standard brain

The parcellation results were stable and consistent on three different datasets (Fig. 5 a), with high reproducibility on each dataset (NMI = 0.75, 0.81 and 0.84, respectively for dataset 1, 2 and 3) and high consistency between the MPMs of different datasets [NMI = 0.92 (for datasets 1 vs. 2), 0.69 (for datasets 1 vs. 3) and 0.71 (for datasets 2 vs. 3)]. Besides, the parcellation results also showed high concentration on the probability maps (Fig. 5b) and resulted in a high coverage fraction for the overlapping areas (i.e., 94 %).

To evaluate the smoothing effects on brain parcellation, we also partitioned MFC on four differently smoothed datasets. As shown in Fig. 5c, highly consistent parcellation results were achieved across differently smoothed datasets, including high reproducibility of group parcellation for each level of smoothness (NMI = 0.75, 0.81, 0.81 and 0.85, respectively for unsmoothed, FWHM = 4, 6 and 8 mm) and high consistency of individual parcellation among differently smoothed datasets [NMI = 0.63 (between unsmoothed and FWHM >0), 0.70 (between FWHM = 4 and FWHM >4) and 0.78 (between FWHM = 6 and FWHM = 8)]. The results (illustrated in Fig. 5c) thus demonstrated that different smoothing conditions had little impact on the performance of our method.

#### Parcellation of the parietal operculum

The parietal operculum was parcellated into multiple subregions on multi-site rs-fMRI datasets. Stable and consistent parcellation results were achieved on the three datasets. The parcellation results were well corresponding to the cytoarchitecture subdivisions (Eickhoff et al. 2006a), but an extra cluster for the head representation was separated which was corresponding to the somatotopic organizations (Eickhoff et al. 2007). The following results were based on local time courses, but similar patterns were also presented when whole-brain connectivity patterns were used (see Supplementary Results 1 in Supplementary materials for a detailed explanation).

**Fig. 6:**
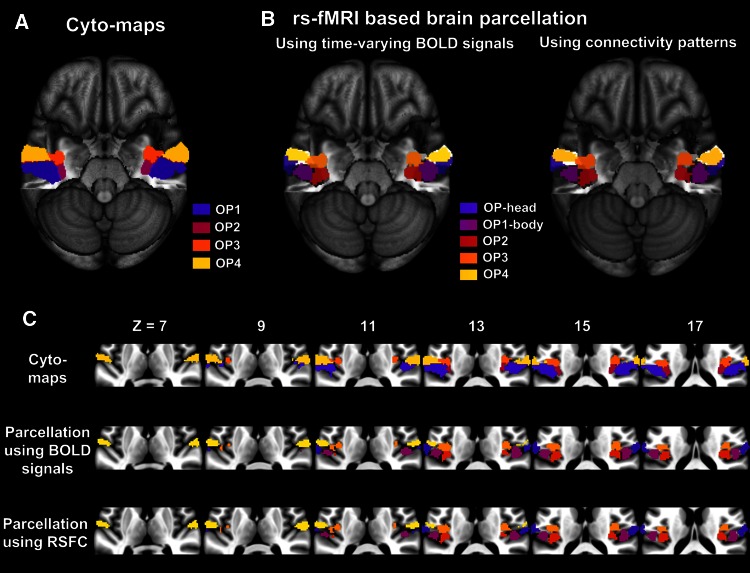
Parcellation of the parietal operculum on multi-site rs-fMRI datasets. Five subregions were identified using rs-fMRI-based brain parcellation (CBP-fMRI). Their overlapping maps across the three datasets were shown in **b**, after a threshold at 50 % on the probability maps of each dataset. Similar patterns were shown using time-varying BOLD signals and whole-brain connectivity patterns. Both of them achieved high consistency across multiple datasets and well correspondence to the cytoarchitectonic subdivisions (cyto-maps), which encompassed four subregions OP1-OP4 (**a**) (Eickhoff et al. 2006a). All these results were rendered by projecting them onto the MNI152 template with the temporal lobes removed to obtain a clear view of the parietal operculum. **c** *Axial slice views* of both cyto-maps and CBP-fMRI results displayed on the MNI152 standard brain

Five stable subregions were identified within the parietal operculum (Fig. 6) (Table S1). High correspondence was achieved as comparing with the cyto-maps (NMI = 0.75, 0.75 and 0.77, respectively for dataset 1, 2 and 3). We renamed the clusters according to their correspondence with the cytoarchitectonic subdivisions (Eickhoff et al. 2006a) and somatotopic organizations (Eickhoff et al. 2007). Specifically, cluster 1, named OP-head, was evenly located at the lateral parts of areas OP1 and OP4, but was corresponding to the head representation area within the somatotopic organizations (Eickhoff et al. 2007). Cluster 2, named OP1-body, was located at the medial part of area OP1, and corresponding to the body representation area in OP1 (Eickhoff et al. 2007). Cluster 5, named OP4, was entirely located within area OP4 and covered most of its territory. The two medial clusters were named as OP2 and OP3, respectively, because they were mainly located within areas OP2 and OP3.

Consistent patterns were presented on the three different datasets (Fig. S5), with high reproducibility on each dataset (NMI = 0.83, 0.85 and 0.85, respectively for dataset 1, 2 and 3), and high consistency between different datasets [NMI = 0.90 (for datasets 1 vs. 2), 0.88 (for datasets 1 vs. 3) and 0.87 (for datasets 2 vs. 3)] (Table S1). The parcellation results shown in Fig. 6b were overlapped among the three datasets after a threshold at 50 % on the probability maps of each dataset, and resulted in a high coverage fraction for the overlapping areas (i.e., 72 %).

## Discussion

In this study, we proposed a sparse representation based method for rs-fMRI-based brain parcellation. We first determined the neighbors for each seed voxel by solving the sparse representation equations and then constructed a sparse similarity matrix based on the representation coefficient matrix to cluster the seed region into separate clusters. We validated the robustness of this approach to brain parcellation, including the ability of restraining noise on simulation data and the consistency of parcellation on real rs-fMRI data. For the simulated rs-fMRI data, we identified the stable sparsity parameter range for the method and showed its consistent high performance on different noisy datasets. For the real rs-fMRI data, stable parcellation of MFC and OP was achieved on three different datasets with high reproducibility within each dataset and high consistency across multiple datasets. The parcellation of MFC was little influenced by different spatial smoothing conditions. Furthermore, the consistent parcellation of OP on multi-site datasets was well corresponding to the cytoarchitectonic subdivisions and their somatotopic organizations.

### Robust brain parcellation using rs-fMRI data

Many parcellation procedures have been proposed for rs-fMRI-based brain parcellation using different similarity measures such as cross-correlation (cc) (Chang et al. 2013; Kim et al. 2010), eta2 (Nelson et al. 2010; Kelly et al. 2012), spatially constrained (sp-local) (Craddock et al. 2012) and Gaussian-kernel weighted on the local time-series matrix (local) (Shen et al. 2010), KNN graph built on the local matrix (KNN) (Shen et al. 2010) and so on. But such parcellation results may be susceptible to various artifacts in rs-fMRI data. Sparse representation, on the other hand, could guarantee a robust brain parcellation. On noisy simulated datasets, sparse representation achieved higher accuracy of parcellation and higher ability of restraining noise effects (Fig. 4). The first three common parcellation methods, including cc, eta2 and sp-local, were quite sensitive to noise and might require high degree of smoothness in real data (i.e., corresponding TSNR of smoothing kernel FWHM = 6 and 8 mm in Fig. 2). Better performance was achieved by the local and KNN methods, but still not as good as our method, especially on highly noisy datasets (i.e., SD = 80 and 100) (Fig. 4). On real rs-fMRI data, sparse representation achieved stable individual parcellation results and consistent group parcellation on multi-site datasets. The probability maps of both MFC and OP were highly centralized on all three datasets (Figs. 5b, 6b). And the parcellation of MFC was little influenced by the degrees of spatial smoothing (Fig. 5c, f). In the meanwhile, the inter-subject variability was preserved through the robust individual parcellation results we achieved on each subject. The inter-subject variability has been widely reported in both structure and function of the human brain (Mueller et al. 2013; Rademacher et al. 2001; Zilles and Amunts 2013). But most previous parcellation studies neglected such variability during brain parcellation by averaging the connectivity profiles (Deen et al. 2011; Yeo et al. 2011) or similarity matrices across subjects (Craddock et al. 2012). In this study, the individual variability of the brain parcellation was characterized by the population probabilistic map on each dataset, which counted the percentage among subjects who had the same cluster labels at the specific voxels. For instance, within MFC, large variance was shown at the boundary of SMA and pre-SMA (Fig. 5b, d). Similar variance patterns of parcellation also occurred in OP, especially at the borders between OP1-body and OP4 (Fig. 6).

The robustness of the method was supported by the following features. First, the similarity graph was constructed based on the representation coefficients rather than commonly used correlation. It employs the multivariate regression model to characterize the unique contribution of each variable. In addition to the self-representation model, an extra sparsity constraint on the representation coefficients is emphasized to identify the most relevant variables. As a result, the most relevant variables in the data could be extracted, which identify the nearest subspaces of each point with minimum embedding dimensions (Elhamifar and Vidal 2013; Wang and Xu 2013). In rs-fMRI data, sparse representation presented an intrinsic local neighboring effect due to the averaging effect of BOLD signals. As an illustration (Fig. S1), the Pearson correlation and sparse representation coefficients were calculated for each voxel within MFC on a real rs-fMRI data. As shown in Fig. S1a, most high correlation values (correlation >0.5) were widely spatially distributed across the whole seed region. Only a small portion of strong connections were captured under strong spatial constraints (*d* < 2), which might hurt its ability in detecting individual modules. Similar conclusion was drawn on the simulation data, where the sp-local method was quite sensitive to noise (Fig. 5). On the other hand, the sparse representation coefficients presented a clear local effect that most of the strong connections were located within a small distance (*d* < 5). Thus, the sparse representation possessed the local neighboring effects without explicit spatial constraints.

Besides, the most relevant variables identified by the representation coefficients support the robustness of the method. These coefficients have been used to reduce noise effects (Elhamifar and Vidal 2013; Elad and Aharon 2006) and recover signals (Elad 2010) in image processing. For rs-fMRI data, it has the potential of reducing noisy artifacts in the BOLD signals. As an illustration (Fig. S2), the sparse representation coefficients were employed to recover the original pattern filled with noisy BOLD signals (i.e., SD = 100) in a simulation data. The pattern was mostly recovered using sparse representation with lambda = 0.1 or 1 (Fig. S2b), which was coincided with the stable sparsity parameter range identified in the simulation data (Fig. 3).

### Parcellation of the parietal operculum

The parietal operculum, which is commonly known as the secondary somatosensory cortex (SII), is located ventrally to the primary somatosensory cortex (SI) and extends into the upper bank of the Sylvain fissure. It has been partitioned into four subdivisions using cytoarchitectonic mapping (Eickhoff et al. 2006a), where OP1–OP4 were, respectively, corresponding to the human homologs of primate areas SII and parietal ventral (PV) (Eickhoff et al. 2007; Eickhoff et al. 2008; Eickhoff et al. 2010), as well as the parieto-insular vestibular cortex (PIVC) (Eickhoff et al. 2007; Zu Eulenburg et al. 2012) and the ventral somatosensory area (VS) (Eickhoff et al. 2007). Clear somatotopic organizations of the four subdivisions have also been revealed, which was also similar to the somatotopic organization of SII, PV, and VS in nonhuman primates (Eickhoff et al. 2007). Generally speaking, the face area was located more laterally at the contiguous border between OP1 and OP4, while the body area was located more medially. However, it is still difficult to distinguish these regions using task fMRI or PET because they usually co-activate in a variety of tactile tasks (Keysers et al. 2010; Burton et al. 2008).

In this study, robust parcellation of the parietal operculum has been achieved using rs-fMRI data. Firstly, similar patterns were achieved using local time-varying BOLD signals and whole-brain connectivity patterns (Fig. 6b). Secondly, the parcellation results were consistent across multi-site datasets (Fig. 6b and Fig. S5), with high reproducibility within each dataset and high consistency across different datasets (Table S1). Thirdly, high correspondence was found as comparing with the cyto-maps (see “Results”). Five functional distinguished subregions has been identified, with four of them well corresponding to the cyto-maps in spatial arrangements. Specifically, cluster OP1-body was located at the medial part of area OP1, corresponding to the body representation area in OP1 (Eickhoff et al. 2007); cluster OP4 was entirely located within area OP4; the two medial clusters were mainly located within areas OP2 and OP3, respectively, but also extended into the lateral areas. The additional cluster named OP-head was evenly located at the lateral parts of areas OP1 and OP4, but it was corresponding to the head representation area in the somatotopic organization (Eickhoff et al. 2007).

Our parcellation results were also consistent with previous functional studies (Keysers et al. 2010; Zu Eulenburg et al. 2013; Burton et al. 2008; Eickhoff et al. 2007). Firstly, we successfully distinguished the lateral subregions (OP1 and OP4) and the medial subregions (OP2 and OP3). Areas OP1 and OP4 formed the secondary somatosensory cortex; area OP2 could be the candidate for the human vestibular cortex (Zu Eulenburg et al. 2012; Eickhoff et al. 2006b); area OP3 joined the posterior insula may serve as the primary cortex for pain (Garcia-Larrea 2012). Secondly, we clearly separated areas OP1 and OP4. Area OP1 was more like a somatosensory integrator for multimodal stimuli and activated in a wide range of somatosensory tasks (Mazzola et al. 2012), while area OP4 may play a role in sensory-motor integration processes with dense connection to the premotor cortex (Eickhoff et al. 2010). Significantly different anatomical connectivity patterns and co-activation patterns have been reported (Eickhoff et al. 2010). The RSFC patterns also showed significant differences between clusters OP1-body and OP4 (Fig. S7). Thirdly, the additional separation of cluster OP-head from clusters OP1-body and OP4 was well corresponding to the somatotopic organization of the human parietal operculum (Eickhoff et al. 2007). MEG studies also showed a lateral to medial transition of representations between tongue, hand and foot (Sakamoto et al. 2008). The RSFC patterns also showed that area OP-head showed higher positive connections with the face area in the primary somatosensory cortex, while OP1-body showed stronger connections with the hand and trunk areas in the primary somatosensory cortex (Fig. S6). Besides, the RSFC maps showed that OP-head had more similar connectivity patterns with OP1-body than OP4, which indicate the functional borders of areas OP1 and OP4 being shifted from the cytoarchitectonic borders laterally.

## Conclusion

In the current study, we presented a robust brain parcellation method using rs-fMRI data, which could achieve stable individual parcellation results with high robustness to noise. It provided an efficient approach to construct a sparse similarity matrix through solving sparse representation equations and generated stable individual parcellation with the aid of spectral clustering. Using the proposed method, similar results were generated using local time-varying BOLD signals and whole-brain connectivity patterns. Moreover, the method outperformed commonly used methods with higher robustness to noise on all simulated rs-fMRI datasets. Highly consistent parcellations were achieved on multi-site real rs-fMRI datasets, along with little influence from different smoothing conditions. Therefore, this parcellation framework using sparse representation presented an efficient approach to robust brain parcellation using resting-state fMRI.

## Electronic supplementary material

Below is the link to the electronic supplementary material.

Supplementary material 1 (DOCX 6553 kb)
