# Supplementary material for: Robust brain parcellation using sparse representation on resting-state fMRI
Source: Brain Struct Funct. 2014 Aug 26;220(6):3565–79. doi: 10.1007/s00429-014-0874-x (PMC4575697; doi:10.1007/s00429-014-0874-x)
Supplement: Supplementary file 1 — Supplementary material 1 (DOCX 6553 kb) [file 429_2014_874_MOESM1_ESM.docx]

Robust Brain Parcellation using Sparse Representation on Resting-State fMRI

Yu Zhang^a,b^, Svenja Caspers^c^, Lingzhong Fan^a^, Yong Fan^a,b^,Ming Song^a,b^, Cirong Liu^f^,

Yin Mo^g^, Christian Roski^c^, Simon Eickhoff^c,d^, Katrin Amunts^c,e^ and Tianzi Jiang^a,b,f,*^.

^a^Brainnetome Center, ^b^National Laboratory of Pattern Recognition,Institute of Automation, Chinese Academy of Sciences, Beijing 100190, P. R. China

^c^Institute of Neuroscience and Medicine (INM-1), Research Centre Juelich, 52425 Juelich, Germany.

^d^Institute for Clinical Neuroscience and Medical Psychology, Heinrich-Heine-University Düsseldorf, 40225 Düsseldorf, Germany

^e^C. and O. Vogt Institute for Brain Research, Heinrich-Heine-University Düsseldorf, 40225 Düsseldorf, Germany

^f^The University of Queensland, Queensland Brain Institute, QLD 4072, Australia

^g^The First Affiliated Hospital of Kunming Medical University, Kunming 650032, P. R. China

**^*^Correspondence Address:**

Prof. Tianzi Jiang, Brainnetome Center, National Laboratory of Pattern Recognition, Institute of Automation, Chinese Academy of Sciences, Beijing, 100190, China

Email: [jiangtz@nlpr.ia.ac.cn](mailto:jiangtz@nlpr.ia.ac.cn) Telephone: +86(0)10 8261 4469; Fax: +86(0)10 6255 1993

# List of Supplementary Materials

## Supplementary texts

#### Methods : Resting-state functional connectivity

#### Results 1: Brain parcellation using RSFC as features

#### Results 2: Brain parcellation using different sparsity parameters

#### Results 3: Hierarchical consistency of OP parcellation

#### Results 4: Functional connectivity patterns of OP subregions

## Supplementary tables

#### Table S1 The performance indicators of OP parcellation

## Supplementary figures

#### Fig. S1 Comparison of the local neighboring effects for the correlation and sparse representation coefficients.

#### Fig. S2 Reconstructing BOLD signals on a noisy simulation data (SD=100) using sparse representation.

#### Fig. S3 Performance of sparse representation with K = 2 and 3 on the simulation data.

#### Fig. S4 Comparing the performance with common parcellation methods when K = 2 and 3 on simulation data.

#### Fig. S5 Parcellation of the parietal operculum on three different datasets.

#### Fig. S6 Parcellation of the parietal operculum using different sparsity parameters.

#### Fig. S7 Differences in functional connectivity of areas OP1 and OP4.

#### Fig. S8 Consistent parcellation of bilateral MFC under different smoothing conditions.

## Supplementary texts

### Methods

#### Resting-state functional connectivity

The functional connectivity pattern of each subregion was generated on each dataset separately. To avoid overlapping among clusters, a ROI was extracted for each subregion on each dataset, with a probability threshold on the population probabilistic map at 50%, and intersected among three datasets. The mean time-course of each ROI was extracted individually and was utilized to calculate the whole-brain functional connectivity using Pearson’s correlation with the normalization using Fisher’s z-transform. Thus, a z-score connectivity map was generated for each cluster and each individual. Finally, the differences in the functional connectivity patterns between subregions were analyzed using the two-factor ANOVA in SPM8, with the first factor including all subjects (N=93) and the second factor corresponding to different subregions. The final results were corrected for multiple comparisons (false discovery rate (FDR) corrected p=0.05, cluster size >30).

### Results

#### Brain parcellation using RSFC as features

Two types of information can be extracted from rs-fMRI datasets for brain parcellation, including time-varying BOLD signals ([Shen et al. 2010](#_ENREF_10); [Goulas et al. 2012](#_ENREF_6)) and functional connectivity profiles ([Kim et al. 2010](#_ENREF_8); [Deen et al. 2011](#_ENREF_3); [Chang et al. 2013](#_ENREF_1); [Kelly et al. 2012](#_ENREF_7); [Nelson et al. 2010](#_ENREF_9)). The rs-fMRI time-courses record the BOLD signals from brain activity, and primarily focus on the temporal similarity between voxels, whereas functional connectivity profiles detect intrinsic functional connectivity within brain networks and emphasize the spatial similarity in functional connectivity patterns. During brain parcellation, both temporal similarities between rs-fMRI time-courses and spatial similarities between functional connectivity profiles can be exploited with similar parcellation results, but the temporal similarities achieved higher stability ([Craddock et al. 2012](#_ENREF_2)).

In our study, similar results were revealed by sparse representation. As shown in Fig. 5, consistent parcellation of MFC were achieved when local time-varying BOLD signals (a-c) or whole-brain functional connectivity patterns (d-f) were used as features. But they also showed certain differences. First, a more vertical boundary between SMA and pre-SMA was identified by using functional connectivity profiles. Second, in comparison to using time-courses as features, lower stability was achieved by using connectivity profiles, including lower reproducibility within each dataset (NMI = 0.73, 0.73 and 0.78, comparing to 0.75, 0.78 and 0.84, respectively for dataset 1, 2 and 3), and higher sensitivity to smoothing conditions (NMI = 0.58, 0.67, 0.71 and 0.75, comparing to 0.75, 0.81,0.82 and 0.85, respectively for unsmoothed, FWHM = 4, 6 and 8 mm).

Similar patterns were also presented for the OP parcellation using whole-brain connectivity patterns on all the three datasets (Fig. 6 and Fig. S4). First, a consistent 5-cluster subdivision were identified on the three datasets, with high reproducibility within each dataset (NMI = 0.78, 0.76 and 0.83, respectively for dataset 1, 2 and 3), and high consistency between different datasets [NMI = 0.83 (for datasets 1 vs 2), 0.76 (for datasets 1 vs 3) and 0.78 (for datasets 2 vs 3)]. Second, high correspondence was also found with the cyto-maps (NMI = 0.66, 0.65 and 0.67, respectively for dataset 1, 2 and 3). The five clusters has the same spatial arrangements as using local time-courses, where cluster OP-head was located at the lateral part of areas OP1 and OP4; cluster OP1-body located at the medial part of area OP1, cluster OP4 covered most territory of area OP4; the two medial cluster mainly located within areas OP2 and OP3 respectively. But, in comparison to using local time-courses, lower stabilities in parcellation were found along with lower agreements with the cyto-maps.

#### Brain parcellation using different sparsity parameters

The sparsity parameter in sparse representation could directly influence the sparsity of the similarity graph and consequently the robustness of the parcellaiton results. On the simulation data, we found that the method cannot restrain high degree of noise when $\lambda$ was too small (i.e. $\lambda<0.1$) and it would fail in pacellation when $\lambda$ was too large (i.e. $\lambda>1$) (Fig. 3). Within the stable parameter range, i.e. the interval [0.1, 1], it could achieve high accuracy of parcellation and high robustness to noise at the same time. On real rs-fMRI data, the parcellation results were mainly based on using $\lambda=1$ during the representation step. Actually, similar parcellation patterns were also achieved by using different lambda values within the stable parameter range. As shown in Fig. S6, consistent patterns of OP parcellation were presented on the third dataset by using different sparsity parameters. Specifically, for all 3-cluster solutions, the parietal operculum was separated into two lateral clusters and one medial cluster, with the two lateral cluster corresponding to OP1 and OP4 respectively, and the medial cluster corresponding to the junction of OP2 and OP3. For all 5-cluster solutions, an extra cluster corresponding to the head area was separated located at the lateral part of areas OP1 and OP4; the medial part of area OP1 was identified as the cluster OP1-body; cluster OP4 covered most territory of area OP4; the two medial cluster mainly located within areas OP2 and OP3 respectively.

#### Hierarchical consistency of OP parcellation

A hierarchical consistency of OP parcellation was captured on all three datasets (Fig. S4). When K = 3, the parietal operculum was separated into two lateral subregions and one medial subregion. The two lateral subregions were corresponding to OP1 and OP4 respectively, and the medial subregion was corresponding to the junction of OP2 and OP3. Despite a little shift of the boundary between lateral and medial subdivisions, our parcellation results showed high correspondence with the cyto-maps on three datasets (NMI = 0.79, 0.78 and 0.80, respectively for dataset 1, 2 and 3) (see ***Table S1*** for other indicators). When K increased from 3 to 5, a clear hierarchical parcellation pattern was presented. The medial subregion corresponding to areas OP2 and OP3 were also separated. An extra cluster corresponding to the head area was separated from lateral part of areas OP1 and OP4, while the medial part of area OP1 remained as cluster OP1-body corresponding to the body area in the somatotopic organization ([Eickhoff et al. 2007](#_ENREF_4)). They also achieved high correspondence with the cyto-maps (NMI = 0.75, 0.75 and 0.77, respectively for dataset 1, 2 and 3).

#### Functional connectivity patterns of OP subregions

We compared the functional connectivity patterns of the subregions corresponding to areas OP1 and OP4. As shown in Fig. S7, cluster OP1-body showed distinct connectivity patterns compared to cluster OP4. Specifically, cluster OP1-body had higher positive connections with the supramarginal gyrus, anterior superior pariatal lobe and inferior frontal gyrus bilaterally, while cluster OP4 had stronger connections with bilateral ventral premotor cortex. Furthermore, cluster OP-head showed higher positive connections with the face area in the primary somatosensory cortex while cluster OP1-body showed stronger connections with the hand and trunk areas in the primary somatosensory cortex. The RSFC maps also showed that OP-head had more similar connectivity patterns with OP1-body than OP4. It might indicate the functional borders of areas OP1 and OP4 shifted from the cytoarchitectonic borders laterally.

## References

Chang LJ, Yarkoni T, Khaw MW, Sanfey AG (2013) Decoding the role of the insula in human cognition: functional parcellation and large-scale reverse inference. Cereb Cortex 23 (3):739-749. doi:10.1093/cercor/bhs065

Craddock RC, James GA, Holtzheimer PE, 3rd, Hu XP, Mayberg HS (2012) A whole brain fMRI atlas generated via spatially constrained spectral clustering. Hum Brain Mapp 33 (8):1914-1928. doi:10.1002/hbm.21333

Deen B, Pitskel NB, Pelphrey KA (2011) Three systems of insular functional connectivity identified with cluster analysis. Cereb Cortex 21 (7):1498-1506. doi:10.1093/cercor/bhq186

Eickhoff SB, Grefkes C, Zilles K, Fink GR (2007) The somatotopic organization of cytoarchitectonic areas on the human parietal operculum. Cereb Cortex 17 (8):1800-1811. doi:10.1093/cercor/bhl090

Eickhoff SB, Schleicher A, Zilles K, Amunts K (2006) The human parietal operculum. I. Cytoarchitectonic mapping of subdivisions. Cereb Cortex 16 (2):254-267. doi:10.1093/cercor/bhi105

Goulas A, Uylings HB, Stiers P (2012) Unravelling the intrinsic functional organization of the human lateral frontal cortex: a parcellation scheme based on resting state FMRI. J Neurosci 32 (30):10238-10252. doi:10.1523/JNEUROSCI.5852-11.2012

Kelly C, Toro R, Di Martino A, Cox CL, Bellec P, Castellanos FX, Milham MP (2012) A convergent functional architecture of the insula emerges across imaging modalities. Neuroimage 61 (4):1129-1142. doi:10.1016/j.neuroimage.2012.03.021

Kim JH, Lee JM, Jo HJ, Kim SH, Lee JH, Kim ST, Seo SW, Cox RW, Na DL, Kim SI, Saad ZS (2010) Defining functional SMA and pre-SMA subregions in human MFC using resting state fMRI: functional connectivity-based parcellation method. Neuroimage 49 (3):2375-2386. doi:10.1016/j.neuroimage.2009.10.016

Nelson SM, Cohen AL, Power JD, Wig GS, Miezin FM, Wheeler ME, Velanova K, Donaldson DI, Phillips JS, Schlaggar BL, Petersen SE (2010) A parcellation scheme for human left lateral parietal cortex. Neuron 67 (1):156-170. doi:10.1016/j.neuron.2010.05.025

Shen X, Papademetris X, Constable RT (2010) Graph-theory based parcellation of functional subunits in the brain from resting-state fMRI data. Neuroimage 50 (3):1027-1035. doi:10.1016/j.neuroimage.2009.12.119

#### Table S1 The performance indicators of OP parcellation.

We evaluated the performance of the parcellation results of the parietal operculum by calculating the reproducibility within each dataset, the consistency across multi-site datasets and the agreement with the cyto-maps ([Eickhoff et al. 2006](#_ENREF_5)). All indicators were calculated using the NMI score (Eq.4)

| **Cluster numbers** | **Datasets** | **Performance indicators** | | |
| --- | --- | --- | --- | --- |
|  |  | **group reproducibility** | **group consistency** | **agreement with cyto-maps** |
| **k=2** | Dataset 1 | 0.84 | 0.91 | 0.77 |
|  | Dataset 2 | 0.79 | 0.95 | 0.76 |
|  | Dataset 3 | 0.81 | 0.91 | 0.78 |
| **k=3** | Dataset 1 | 0.80 | 0.92 | 0.79 |
|  | Dataset 2 | 0.73 | 0.86 | 0.78 |
|  | Dataset 3 | 0.85 | 0.86 | 0.80 |
| **k=4** | Dataset 1 | 0.69 | 0.78 | 0.74 |
|  | Dataset 2 | 0.79 | 0.82 | 0.72 |
|  | Dataset 3 | 0.78 | 0.72 | 0.74 |
| **k=5** | Dataset 1 | 0.83 | 0.90 | 0.75 |
|  | Dataset 2 | 0.85 | 0.88 | 0.75 |
|  | Dataset 3 | 0.85 | 0.87 | 0.77 |
| **k=6** | Dataset 1 | 0.77 | 0.89 | 0.76 |
|  | Dataset 2 | 0.72 | 0.87 | 0.77 |
|  | Dataset 3 | 0.83 | 0.87 | 0.77 |


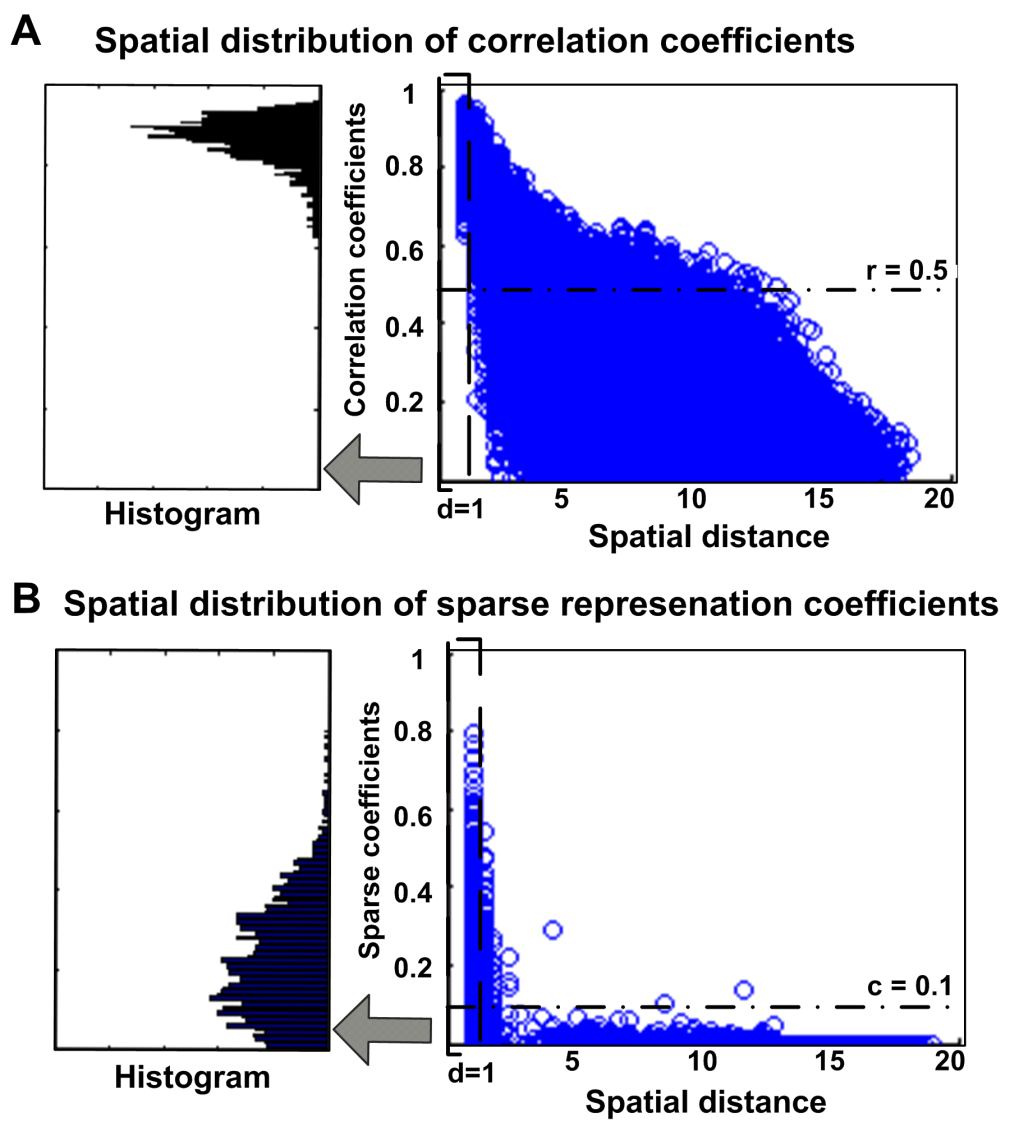


#### Fig. S1 Comparison of the local neighboring effects for the correlation and sparse representation coefficients.

The spatial distributions of the correlation coefficients were shown (a). A threshold (r_ij_ >=0.5) was performed on the correlation coefficients to show its widely spatial distribution. The histogram of the correlation coefficients within the arrow box (d_ij_ <=1) was shown in the left column. The spatial distributions of the sparse representation coefficients were shown (b). Most sparse coefficients (c_ij_ >=0.1) were located within a small spatial neighborhood. The histogram of the correlation coefficients within the arrow box (d_ij_ <=1) was shown in the left column.


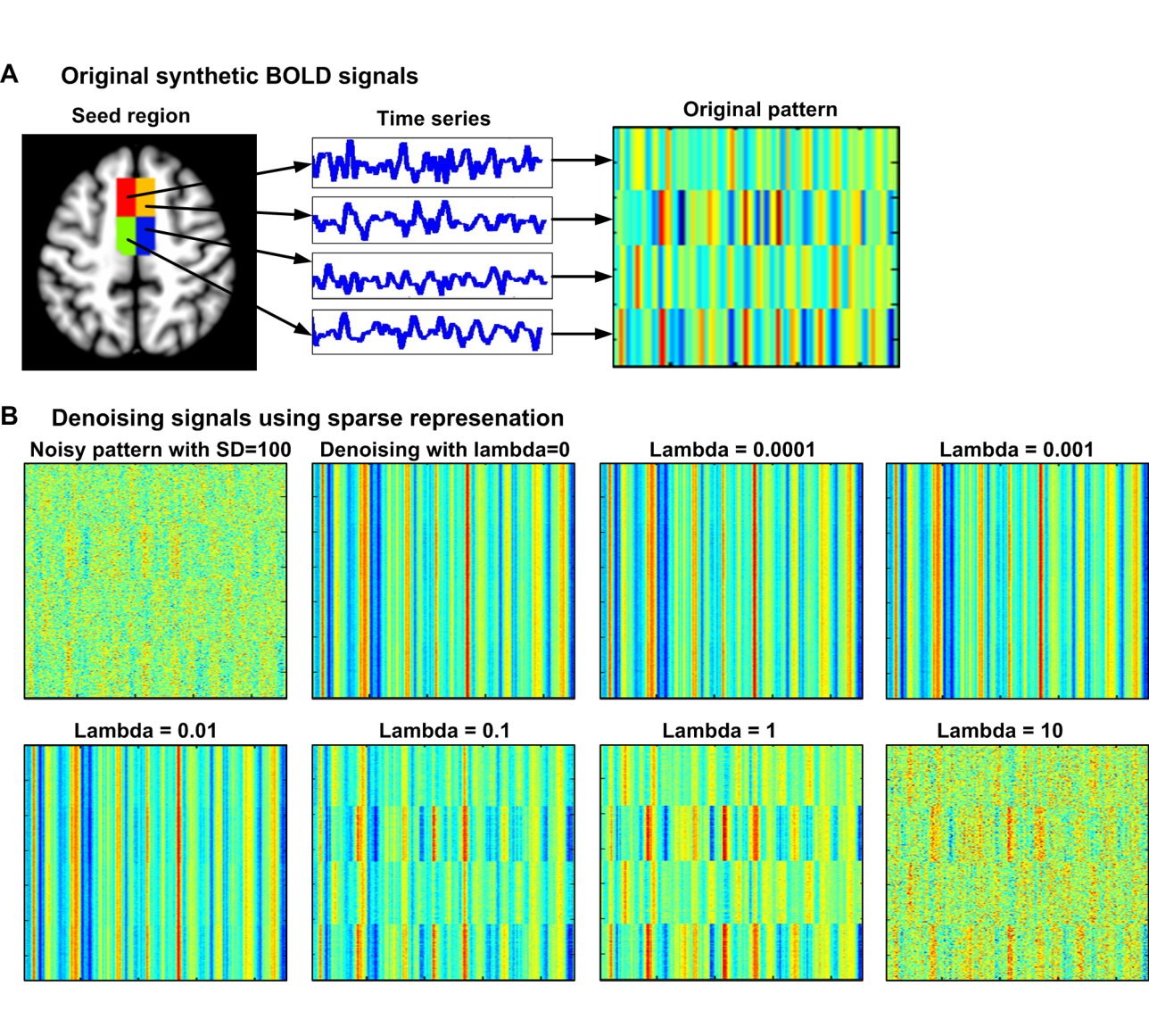


#### Fig. S2 Reconstructing the patterns of synthetic BOLD signals on a noisy simulation data (SD=100) using sparse representation.

The original pattern was constructed by filling each of the four subunits within medial frontal cortex with corresponding synthetic BOLD signals (a). Starting with the noisy pattern which included high amount of Gaussian noise (SD = 100), the synthetic pattern was denoised by using the sparse representation coefficients of different sparsity parameters (b). The results showed that sparse representation with lambda = 0.1 and 1 had high ability of recovering signals.


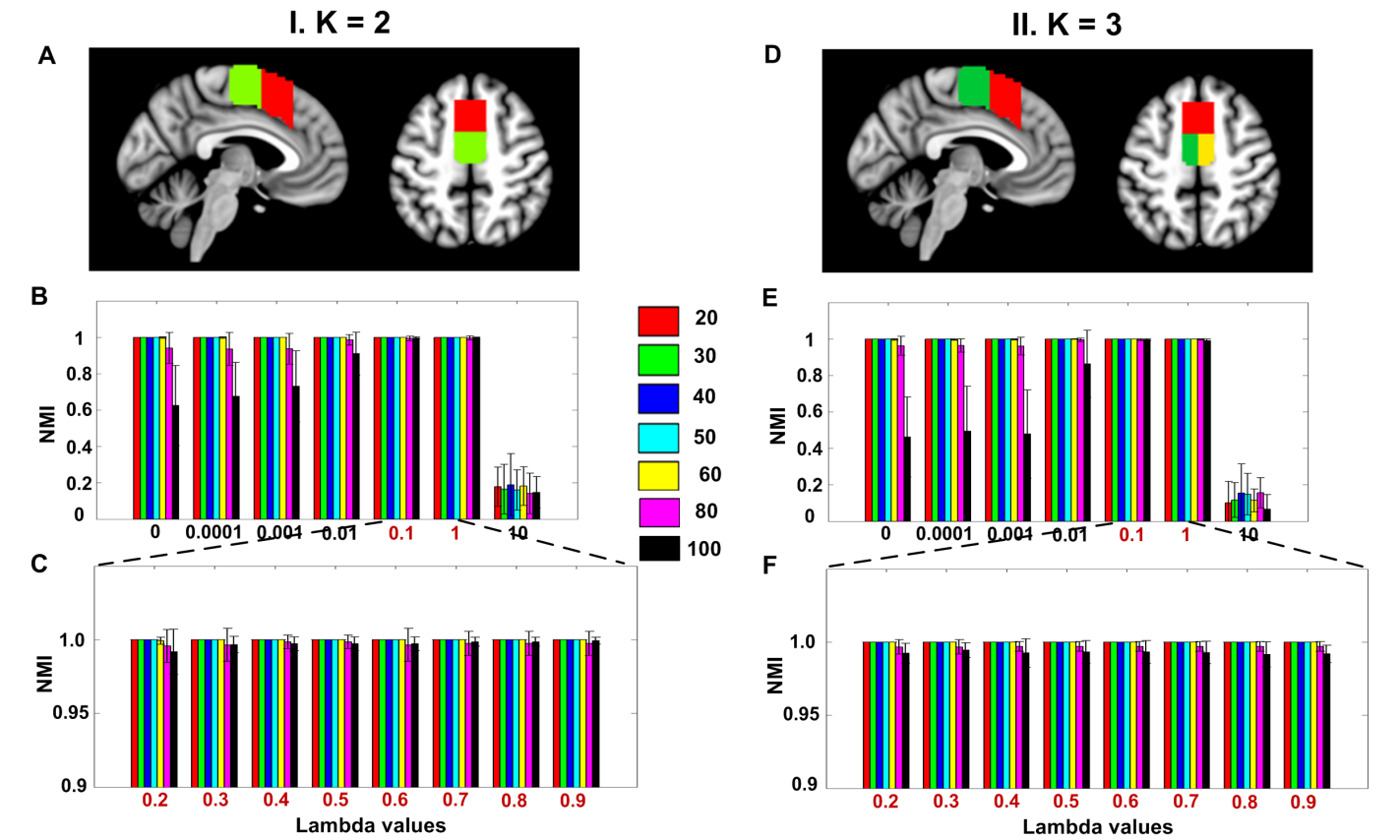


#### Fig. S3 Performance of sparse representation with K = 2 and 3 on the simulation data.

Sparse representation was tested on multiple noisy simulated datasets with two $\lambda$ sequences. (a)-(c) showed the results with K = 2 and (d)-(f) showed the results with K = 3. A stable sparsity parameter range labeled with the red color were identified on both cases, which achieved highly stable performance on all noisy datasets. The mean NMI scores across ten subjects was used to evaluate the accuracy of parcellation, with different colors indicating different noisy datasets, i.e. SD(noise) = 20, 30, 40, 50, 60, 80 and 100, and the error bars representing the SD of NMI values.


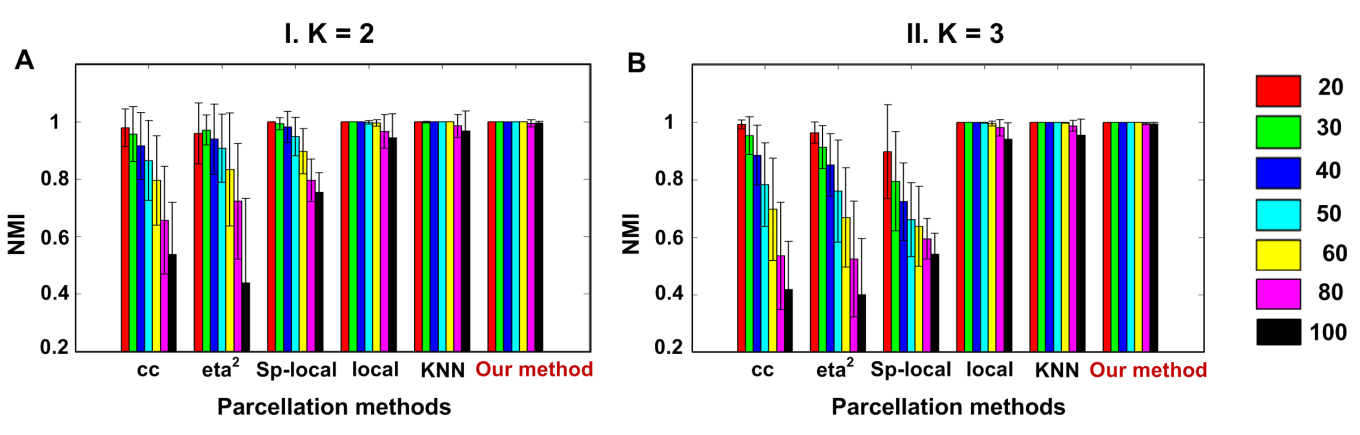


#### Fig. S4 Comparing the performance with common parcellation methods when K = 2 and 3 on simulation data.

Five commonly used similarity matrices were tested and compared on different noisy datasets, i.e. SD(noise) = 20, 30, 40, 50, 60, 80 and 100. For both K = 2 (a) and 3 (b), our method achieved higher performance than all common methods on highly noisy data (SD = 80 and 100) and showed higher robustness to noise. The accuracy of parcellation was evaluated through a comparison with the ground truth using normalized mutual information (NMI). cc: cross-correlation; local: using local time-varying BOLD signals; Sp-local: performing spatially constraints on the local matrix; KNN: constructing a KNN graph on the local matrix; Our method: performing sparse representation on local time-varying BOLD signals.


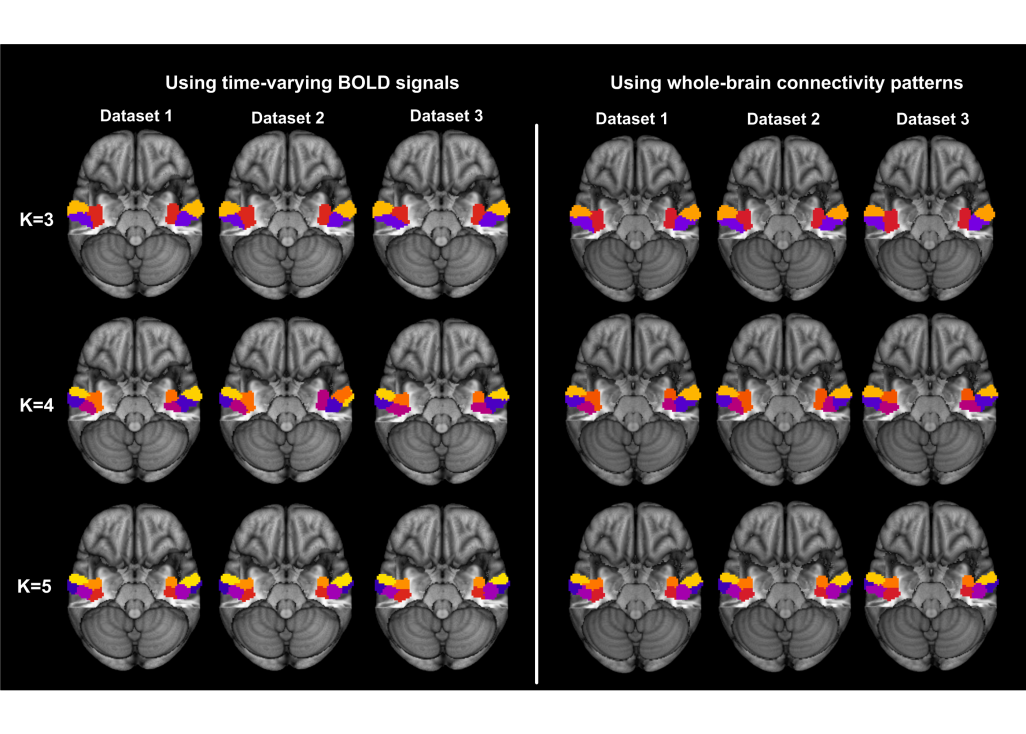


#### Fig. S5 Parcellation of the parietal operculum on three different datasets.

Consistent parcellation of the parietal operculum were achieved on the three rs-fMRI datasets with the cluster number increased from 3 to 5. Similar patterns were presented by using the time-varying BOLD signals and whole-brain connectivity patterns. All results were projected onto the MNI152 template with the temporal lobes removed to obtain an clear view of the parietal operculum.


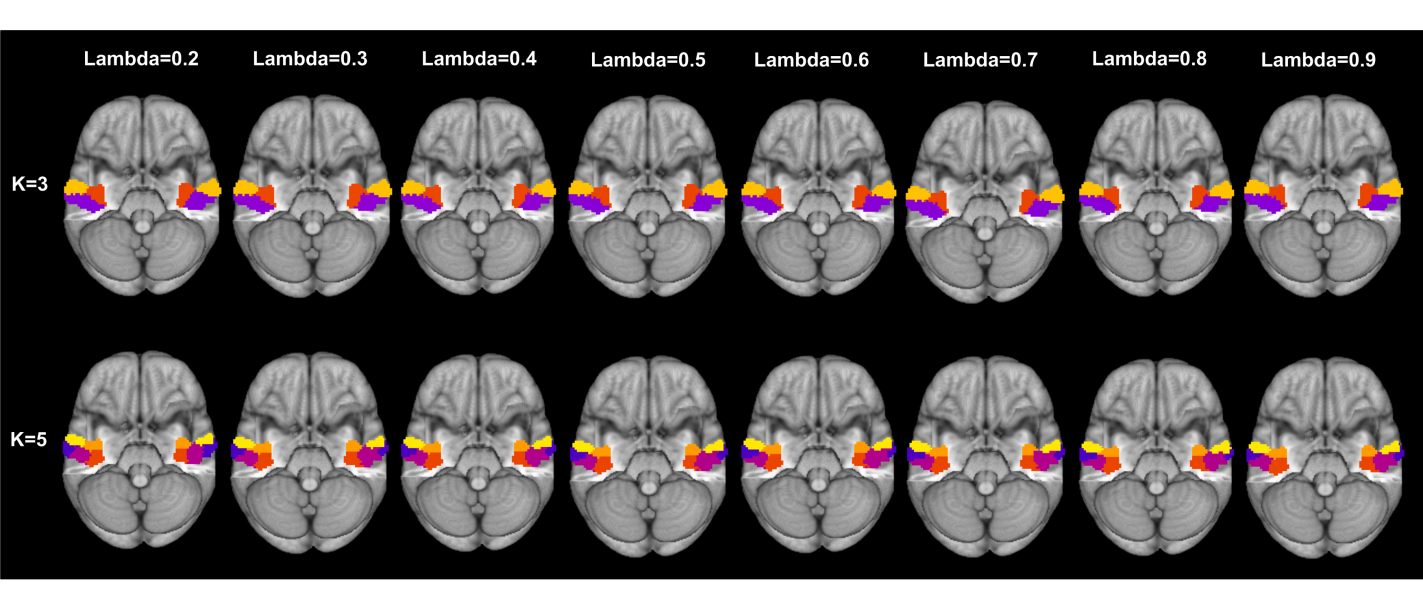


#### Fig. S6 Parcellation of the parietal operculum using different sparsity parameters.

Consistent patterns were presented by using different stable sparsity parameters in sparse represenation for all 3- and 5-cluster solutions. Only results on the third datasets were shown.


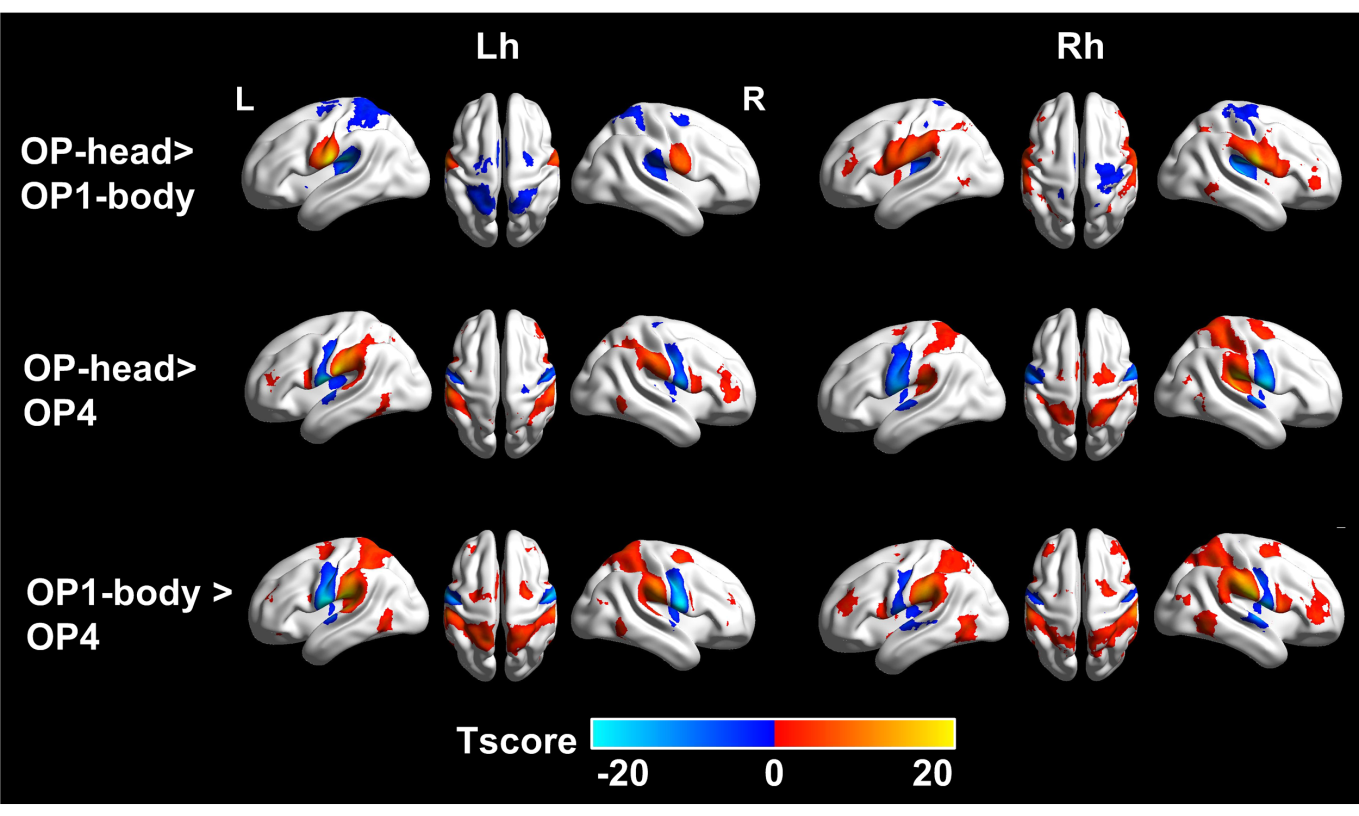


#### Fig. S7 Differences in functional connectivity of areas OP1 and OP4.

The differences in connectivity patterns were analyzed using ANOVA on the positive functional connectivity maps of each subregion and corrected for multiple comparisons (FDR p=0.05, cluster size > 30). They were projected on a 3D brain surface for visualization using the BrainNet Viewer (http://www.nitrc.org/projects/bnv/ ).


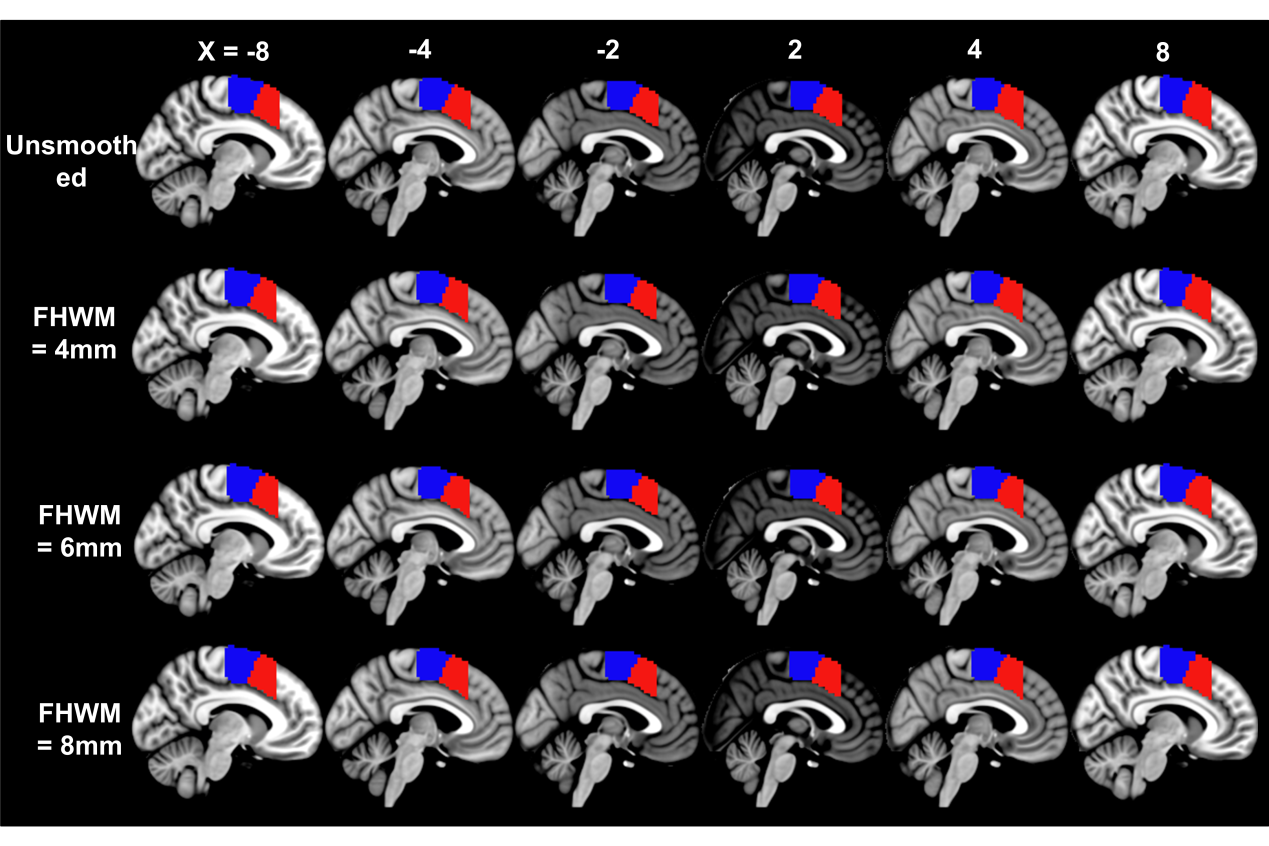


#### Fig. S8 Consistent parcellation of bilateral MFC under different smoothing conditions.

Multislice view of the consistent parcellation of bilateral MFC under different smoothing conditions, i.e. unsmoothed, FWHM = 4, 6 and 8 mm. Only parcellation results using time-varying BOLD signals were shown here, but similar patterns were also achieved by using whole-brain connectivity patterns.
